# Use of extracorporeal membrane oxygenation for treating acute cardiomyopathy after liver transplantation: a case report

**DOI:** 10.5935/0103-507X.20180029

**Published:** 2018

**Authors:** Rodrigo Santos Biondi, Vitor Salvatore Barzilai, André Luis Conde Watanabe, Gustavo de Sousa Arantes Ferreira, Fernando Antibas Atik

**Affiliations:** 1 Unidade de Terapia Intensiva Cirúrgica, Instituto de Cardiologia do Distrito Federal - Brasília (DF), Brasil.; 2 Unidade de Transplante Hepático, Instituto de Cardiologia do Distrito Federal - Brasília (DF), Brasil.; 3 Departamento de Cirurgia Cardíaca, Instituto de Cardiologia do Distrito Federal - Brasília (DF), Brasil.

**Keywords:** Liver transplantation, Extracorporeal membrane oxygenation, Shock, cardiogenic, Cardiomyopathies, Coagulation agents, Case reports

## Abstract

We report the case of a female patient, 58 years of age, without known heart disease, who underwent liver transplantation without complications. On the second postoperative day, the patient developed cardiogenic shock secondary to stress-induced cardiomyopathy (Takotsubo-like syndrome). The patient was successfully managed with veno-arterial peripheral extracorporeal membrane oxygenation for 6 days, with complete recovery of cardiac function and of the hepatic graft. Coronary syndrome and acute myocarditis were excluded as the causes of the shock. The use of extracorporeal membrane oxygenation in this scenario is possible and safe, considering its specialized protocols and treatment.

## INTRODUCTION

The postoperative period of liver transplantation presents challenges associated with clotting management (where bleeding due to thrombocytopenia, *hypofibrinogenemia* and clotting factor deficiency is frequent), liver graft function management (risk of thrombosis of liver vessels and rejection) and hemodynamic management.

Cardiac complications such as acute pulmonary edema, myocardial ischemia and tachyarrhythmia are relatively common after liver transplantation, particularly in elderly patients and critical recipients.^(^^1^^)^ However, cardiogenic shock is less frequently observed during the postoperative period and may be related to subclinical coronary disease that was not detected in the preoperative period, ventricular arrhythmias, pulmonary embolism, or new cardiomyopathy - whether caused by myocarditis,^(^^2^^)^ cirrhotic cardiomyopathy^(^^3^^)^ or extrahepatic iron deposition.^(^^4^^)^

In the present study, we report cardiogenic shock secondary to stress-induced cardiomyopathy after liver transplantation that was successfully treated with mechanical extracorporeal support, with full recovery of cardiac function. 

## CASE REPORT

A female patient, 58 years of age, presenting with cryptogenic cirrhosis, underwent liver transplantation from a cadaver donor on 24 June 2015. During the preoperative period, the patient did not exhibit any alterations of other organs or systems nor cardiovascular comorbidities. She was subjected to routine cardiac assessment through pharmacologic stress echocardiography (ECHO), which was considered normal (ejection fraction - EF: 71.45%).

The perioperative period occurred without complications, and the patient did not receive blood-derived products. After reperfusion, she presented with acute atrial fibrillation, which was successfully reversed with administration of metoprolol. The organ cold ischemia time lasted 8 hours and 40 minutes.

The patient was then transferred to the intensive care unit (ICU) intubated, in use of noradrenaline at 0.15mcg/kg/minute (arterial pressure - AP: 116 x 67mmHg), under sinus rhythm (heart rate - HR 90bpm). She exhibited good diuresis through the urine collector, with good peripheral perfusion. She was extubated approximately 3 hours after admission. Early during the first postoperative day, the patient presented with an episode of atrial fibrillation that required reintubation even after the reversal of arrhythmia. There was a significant drop in hemoglobin (from 9.5g/dL to 6.5g/dL) and expressive abdominal distension, but with no signs of shock. A computed tomography (CT) scan of the abdomen was performed, which showed signs of hemoperitoneum and ischemia of the hepatic segments V/VI. She was then subjected to exploratory laparotomy, which revealed good liver perfusion, blood in the cavity and blood clots with no areas of active bleeding. The hypothesis of compartment syndrome was raised due to the tension of the abdominal wall closure associated with the recent worsening of kidney function, which led to the use of mesh wrapping to allow closure without pressure.

Despite the improvement in hemodynamics, the clinical course evolved to oliguric acute renal failure and a requirement for hemodialysis with important acidosis and the establishment of continuous hemodialysis (continuous venovenous hemofiltration). 

On the second postoperative night, the patient presented with progressive hypotension and shock (mean arterial pressure MAP: 54mmHg; with noradrenaline: 0.9mcg/kg/minute; vasopressin: 0.02 Ui/minute; dobutamine: 15mcg/kg/minute; lactate: 63mg/dL and 53% saturated venous oxygen - SVO_2_), with signs of elevated of the filling pressure through qualitative ECHO at the bedside. There were no signs of myocardial ischemia, and the serial electrocardiogram (ECG) and troponin were normal. Transthoracic ECHO showed severe dysfunction of the left ventricle (EF: 12%), with severe distension of the left ventricle, "diffuse hypokinesia and dyskinesia of the apex" and a 48.31mL/m^2^ indexed volume of left atrium, in addition to a peak tricuspid speed of 284 cm/s, which confirmed the previous impression of high filling pressure highlighted by the intensivist on bedside ECHO. There were no signs of pericardial effusion.

The etiological possibility of catecholaminergic cardiomyopathy or fulminant myocarditis was raised. Due to the highly severe cardiogenic shock of rapid evolution and risk of death, the team decided to install venoarterial extracorporeal membrane oxygenation (ECMO). The procedure was performed at 2 AM on June 27 by the cardiac surgeon, in the ICU, through puncture of the right femoral artery and the left femoral vein with an initial flux of 4.5L/minute.

The patient had a good response after the installation of the circulatory support with recovery of perfusion. The vasopressors and inotropes were weaned in a few hours. After 6 hours of circulatory support, the patient had a MAP of 60mmHg without the need for noradrenaline and dobutamine at 5mcg/kg/minute. Dobutamine was discontinued on the next day. Continuous venovenous hemofiltration was maintained for metabolic control.

The patient presented with signs of coagulopathy prior to installing the ECMO (prothrombin activity time - PT between 20.2 and 25.2%, and platelets between 62,000 and 33,000, decreasing). During ECMO, thrombocytopenia persisted (with approximately 30,000 platelets), likely due to hepatic dysfunction associated with shock. Constant monitoring of membrane oxygenation and clotting was implemented. Anticlotting therapy was not started during this period,^(^^5^^)^ and no signs of blood clots or damage were observed on the oxygenator performance. Despite the coagulopathy, there were no new events associated with the retroperitoneal hematoma that was drained earlier. Immunosuppression was initiated according to the institutional protocol (methylprednisolone, tacrolimus and mycophenolate sodium). On 26 June 2015, immunosuppression was suspended as a result of the shock. Starting on 28 June 2015, tacrolimus was reintroduced and steroids were used to maintain immunosuppression until the patient was discharged from the ICU.

There was a progressive recovery of myocardial function, and the ECMO was successfully weaned after 6 days with 36% EF and no bleeding or thrombosis events. 

Hepatic and cardiac functions were completely restored in approximately 30 days (Table 1). The patient was discharged from the ICU on 22 July 2015 while still under hemodialysis, but she presented gradual recovery of renal function. The last hemodialysis session was on 22 September 2015. Prior to hospital discharge, the patient was in better condition, and acute myocarditis and coronary heart disease were ruled out through nuclear magnetic resonance and coronary angiotomography, respectively.

**Table 1:** Laboratory exams and hemodynamic profile

	IPP	1º PP	2º PP*	3º PP†	4º PP	5º PP	6º PP	7º PP	8º PP	9º PP‡	28º PP
	24/6/15	25/6/15	26/6/15	27/6/15	28/6/15	29/6/15	30/6/15	1/7/15	2/7/15	3/7/15	22/7/15
Hemoglobin	9.50	6.50	9.00	7.70	9.80	10.50	11.00	10.00	10.20	9.00	9.10
Platelets	55,500	49,600	33,000	18,000	25,000	28,000	29,000	43,000	32,000	57,000	97,000
AST	639	926	631	218	110	78	60	61	100	99	45
ALT	315	404	540	238	187	142	106	85	104	115	57
Total bilirubin	3.40	1.50	1.80	2.00	1.50	1.60	1.20	1.30	1.00	0.80	0.50
PT (%)	16.6	20.2	24.8	47.0	67.1	71.0	81.9	96.0	99.0	85.0	53.0
Fibrinogen	62.00	66.00	75.00	76.00	96.00	105.00	231.00	233.00	213.00	218.00	320.00
Noradrenaline (mcg/kg/minute)§	0.15	0.40	0.90	0.05	--	--	--	--	--	--	--
Dobutamine (mcg/kg/minute)§	--	--	20	5	--	5	--	--	--	--	--
EF (%)	--	--	12	--	--	25	--	29	--	36	54
ECMO flow (L/minute)	--	--	--	4.50	4.10	4.10	2.1	2	2	1.2	--

IPP - immediate postoperative period; PP - postoperative period; AST - aspartate transaminase; ALT - alanine transaminase; PT - prothrombin activity time; EF - ejection fraction; ECMO - extracorporeal membrane oxygenation.

* ECMO preinstallation;

† in ECMO;

‡ ECMO predecannulation/withdrawal;

§ maximum dose used.

The patient was discharged under Functional Class I (New York Heart Association -NYHA) in September 2015 and, at present, continues to be under routine evaluation by the liver transplantation team, with no cardiac event being observed since that time.

This case report was approved by the Ethics Committee of the Institute of Cardiology of the Federal District, protocol (CAAE) 57074916.4.0000.0026.

## DISCUSSION

This case report describes a patient who developed cardiogenic shock 2 days after liver transplantation through a suggestive stress-induced cardiomyopathy, catecholaminergic or Takotsubo-like syndrome.^(^^6^^)^ Both the transplant and the laparotomy for the hemoperitoneum may have triggered this syndrome.

Takotsubo syndrome is characterized by a transient left ventricular dysfunction, predominantly medioapical, that is usually triggered by physical or emotional stress. Although its pathophysiology is still not completely understood, emotional stress seems to play a predominant role through catecholaminergic discharge. The diagnosis requires coronary angiography and ventriculography to rule out coronary syndrome. More modern approaches have proposed diagnosis by non-invasive methods, incorporating data from ECG, echocardiography and magnetic resonance imaging studies.^(^^7^^)^

All other causes of acute cardiac failure post-liver transplantation were ruled out. A comprehensive preoperative evaluation was carried out where any subclinical condition was rejected and where no cardiac manifestation was observed. This excluded the possibility of cirrhotic cardiomyopathy, which is well detected though pharmacological stress ECHO.^(^^3^^)^ Acute coronary syndrome was also ruled out based on electrocardiograms and myocardial necrosis markers, which were negative.

This case shows that the cause of acute heart failure (catecholaminergic), even in the context of liver transplantation, should not be underestimated. In the literature, there are at least three other reports of this syndrome after liver transplantation,^(^^6^^,^^8^^)^ which occurred with heart failure and cardiogenic shock but was successfully treated with pharmacological management only. A retrospective review of 1,460 transplants in a single medical center detected 17 cases of non-ischemic cardiomyopathy.^(^^9^^)^ The present case report was the first case in this center and differs from other reported cases due to the need for extracorporeal circulatory support, which was crucial in treating the cardiogenic shock, as well as in the complete restoration of liver and heart function. It is known that short-term mechanical circulatory assistance such as the ECMO yields excellent responses in Takotsubo syndrome cases.

There are no reports in the literature of ECMO use in this context. However, the use of mechanical circulatory support with biventricular assist device post-liver transplantation was successful in a case of cardiac dysfunction associated with hemochromatosis. The biggest challenges in these cases consist of bleeding control and decisions about anticoagulation therapy, which is needed to maintain the viability of membrane oxygenation, in patients with coagulopathy associated with the underlying disease and transplant. Despite the complexity of extracorporeal ventricular assistance and the risk of adding morbidity in patients with a severe clinical case, experimental data have shown that this is a safe procedure, with no significant differences in respiratory, metabolic and hemodynamic variables observed before and after the contact of blood with the ECMO circuit and priming.^(^^10^^)^

In critical scenarios such as this with a high risk of death and a likely reversible cause of cardiomyopathy, the only option is short-term circulatory support. The ease of its installation at the bedside, with rapid re-establishment of the arterial flow, has demonstrated ECMO to be the ideal choice for mechanical circulatory support for acute cardiac failure (INTERMACS 1). This approach can be used either as a bridge to recovery or for making decisions. The role of the team, experienced in the technique, and well-designed protocols are essential to the outcome in these complex environments.

Because it is a high-cost resource, the decision on the implant involves well-defined parameters such as the real benefit and contemplation of a tangible clinical recovery rationale. The patient in this case report had thorough recovery conditions, considering the most likely etiology of cardiogenic shock.

## CONCLUSION

The use of extracorporeal membrane oxygenation for circulatory support as a strategy for recovery may be considered an alternative approach to fulminant heart failure of reversible cause after liver transplantation.
